# Spatially Explicit Analysis of Genome-Wide SNPs Detects Subtle Population Structure in a Mobile Marine Mammal, the Harbor Porpoise

**DOI:** 10.1371/journal.pone.0162792

**Published:** 2016-10-26

**Authors:** Ljerka Lah, Daronja Trense, Harald Benke, Per Berggren, Þorvaldur Gunnlaugsson, Christina Lockyer, Ayaka Öztürk, Bayram Öztürk, Iwona Pawliczka, Anna Roos, Ursula Siebert, Krzysztof Skóra, Gísli Víkingsson, Ralph Tiedemann

**Affiliations:** 1 Unit of Evolutionary Biology/Systematic Zoology, Institute of Biochemistry and Biology, University of Potsdam, Potsdam, Germany; 2 Deutsches Meeresmuseum, Stralsund, Germany; 3 Dove Marine Laboratory, School of Marine Science and Technology, Newcastle University, Cullercoats, North Shields, United Kingdom; 4 Marine Research Institute, Reykjavík, Iceland; 5 North Atlantic Marine Mammal Commission, Tromsø, Norway; 6 Marine Biology Department, Faculty of Fisheries, Istanbul University, Istanbul, Turkey; 7 Hel Marine Station, University of Gdansk, Hel, Poland; 8 Swedish Museum of Natural History, Stockholm, Sweden; 9 Institute for Terrestrial and Aquatic Wildlife Research (ITAW), University of Veterinary Medicine Hannover Foundation, Büsum, Germany; Universita degli Studi della Tuscia, ITALY

## Abstract

The population structure of the highly mobile marine mammal, the harbor porpoise (*Phocoena phocoena*), in the Atlantic shelf waters follows a pattern of significant isolation-by-distance. The population structure of harbor porpoises from the Baltic Sea, which is connected with the North Sea through a series of basins separated by shallow underwater ridges, however, is more complex. Here, we investigated the population differentiation of harbor porpoises in European Seas with a special focus on the Baltic Sea and adjacent waters, using a population genomics approach. We used 2872 single nucleotide polymorphisms (SNPs), derived from double digest restriction-site associated DNA sequencing (ddRAD-seq), as well as 13 microsatellite loci and mitochondrial haplotypes for the same set of individuals. Spatial principal components analysis (sPCA), and Bayesian clustering on a subset of SNPs suggest three main groupings at the level of all studied regions: the Black Sea, the North Atlantic, and the Baltic Sea. Furthermore, we observed a distinct separation of the North Sea harbor porpoises from the Baltic Sea populations, and identified splits between porpoise populations within the Baltic Sea. We observed a notable distinction between the Belt Sea and the Inner Baltic Sea sub-regions. Improved delineation of harbor porpoise population assignments for the Baltic based on genomic evidence is important for conservation management of this endangered cetacean in threatened habitats, particularly in the Baltic Sea proper. In addition, we show that SNPs outperform microsatellite markers and demonstrate the utility of RAD-tags from a relatively small, opportunistically sampled cetacean sample set for population diversity and divergence analysis.

## Introduction

Variation in genetic diversity across space is the result of past and present geographic, ecological, and behavioral barriers to gene flow, yielding locally disparate evolutionary trajectories of mutation, drift, and/or selection [1]. In seemingly continuous marine habitats, lack of population structure or panmixia may occur in marine animals that can traverse vast geographic ranges and have high dispersal potential [2–4]. Highly mobile cetacean species, however, often show genetic and morphological differentiation over smaller geographic scales [5]. The determinants of genetic structure in cetacean species are often complex–local environmental differences, ecological specializations along with complex social and behavioral structure can promote divergence [1,6–8]. For instance, many species are known to migrate seasonally around feeding grounds, but may return to distinct areas for mating and breeding [9–13]. Pelagic and offshore ecotypes have been reported in the Atlantic common bottlenose dolphin (*Tursiops truncatus*) populations [14,15], a hierarchical genetic structure has been observed in the Atlantic spotted dolphin (*Stenella frontalis*) [16], and prey specialization has contributed to genetic differentiation in killer whales (*Orcinus orca*) [17].

Harbor porpoise (*Phocoena phocoena*) populations in Western Palearctic waters offer another example of complex genetic differentiation in a highly mobile cetacean species [8,18,19]. The entire eastern Atlantic population (*P*. *p*. *phocoena*) from the northern Bay of Biscay to the coastal waters of Norway and Iceland is a continuous population with weak structure and significant isolation-by-distance (IBD). It is distinct from the Black Sea population (*P*.*p*. *relicta*), as well as from the harbor porpoises from the Iberian coast and Mauritania [8,19]. Glaciations in the northern hemisphere caused the cooling of the Mediterranean and created a suitable habitat for the expansion of the harbor porpoise. Subsequent climate warming caused habitat fragmentation and the migration of harbor porpoises into the cooler Black Sea, where they now form a relict population [8].

A similar peripheral marine ecosystem, where dispersal is limited, is the Baltic Sea. It is a sub-basin of the Atlantic Ocean formed less than 10,000 years before present (BP) as a postglacial marine environment [20]. The earliest harbor porpoise fossils have been dated to approximately 9000 years BP in the western Baltic [21,22]. From 7500 yr BP, fossils were also identified in the Gulf of Bothnia and Finland [21]. Despite this relatively short history, populations of marine organisms in the Baltic are genetically distinct from conspecifics from the North Sea and the Atlantic, most likely due to isolation, bottlenecks, and—in some cases—local adaptation[23–25]. A series of basins, separated by shallower underwater ridges ranging from the North Sea through Skagerrak, Kattegat, and the Belt Seas (BES) to the entrance to the Baltic Sea proper may hinder gene flow [23,26]. The abundance of harbor porpoises was estimated via line transect surveys at over 40,000 individuals in the Skagerrak, Kattegat, and BES regions [27]. Aerial, acoustic and visual surveys suggest that the population size of harbor porpoises inhabiting the Inner Baltic Sea (IBS) region is two orders of magnitude lower–only several hundred individuals at most [28–31].

Three harbor porpoise populations have been proposed to inhabit the waters between the North Sea and the Inner Baltic Sea; the North Sea/Skagerrak population, the southern Kattegat/BES population, and a population in the IBS, based on morphology and genetic markers (microsatellites and mitochondrial DNA) [32–36]. The most recent large-scale population genetics study [32] indicates subtle population differentiation between the Baltic Sea regions, and partially addresses earlier criticism, which suggested that managing Baltic porpoises as an independent conservation unit is premature [37]. Morphological and tracking studies suggest some overlap in transition zones between geographical regions in the Baltic [33,38]. A recent study addressed the need to establish more reliable population delineation of Baltic harbor porpoises [27]. A further study, which also considered seasonal migrations of these small cetaceans, used acoustic monitoring and satellite tracking to define three management units–in the North Sea, the Belt Sea, and the Baltic Sea proper [39].

The present analysis aims to use genomics techniques to improve population structure resolution within the Baltic Sea and adjacent regions. We investigated population structure using a dataset of samples collected over the range of the harbor porpoise distribution from the Western Black Sea to the North Sea to the Inner Baltic, in order to place the population structure of the Baltic samples in a broader context. We combined double digest restriction site-associated DNA (ddRAD) libraries and high-throughput sequencing on an Illumina platform. Using an explicit spatial analysis and Bayesian clustering, we analyzed our genome-wide SNPs together with previously used molecular markers (microsatellites and mitochondrial Control Region sequences [32]) for the same sample set, in order to assess the gain of resolution obtained by the population genomic approach.

## Materials and Methods

### Geographic coverage, sampling strategy and study design

**Fig 1:**
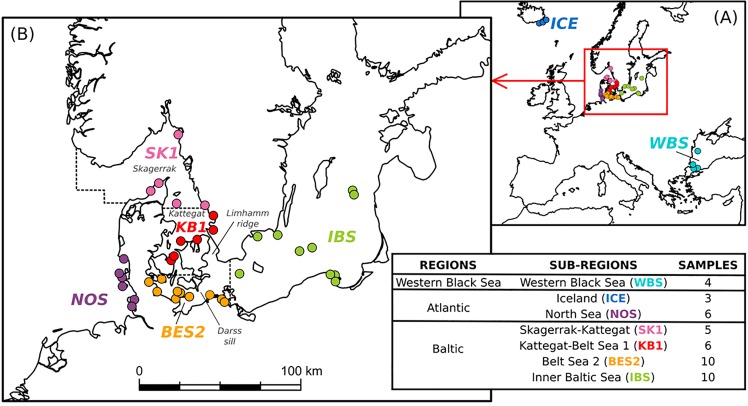
Sampling locations and assignment to geographic sub-regions. (A) A map of European Seas with circles representing collection sites for individual samples from the Western Baltic Sea (WBS), the Atlantic and the Baltic Sea regions. WBS and Icelandic samples (part of the Atlantic region) are labeled with light blue and dark blue circles, respectively. (B) Collection sites for individual samples from the North Sea sub-region (NOS; purple), and Baltic Sea sub-regions: Skagerrak-northern Kattegat (SK1; pink), southern Kattegat-Belt Sea 1 (KB1; red), Belt Sea 2 (BES2; orange), Inner Baltic Sea (IBS; green). Borders between SK1 and KB1, and BES2 and IBS (dashed lines) were based on proposed borders between management units at 56.95°N latitude, and 13.5°E longitude, respectively (39). Borders between NOS and SK1, and KB1 and BES2 are based on [32]. Geographic assignment to regions and sub-regions is summarized in a table (bottom right). Map data: ESRI (2013).

To provide a broad geographic framework for our population genomic study, and in order to place the findings in the context of existing knowledge on harbor porpoise population structure, we selected 44 samples from European seas. For clarity, we refer to three separate geographic regions throughout the manuscript: (1) the western Black Sea region (WBS), (2) the North Atlantic region with the Icelandic (ICE) and North Sea (NOS) sub-regions, and the (3) Baltic region (Fig 1). In the latter, we designated four sub-regions, i.e., SK1, KB1, BES2, and IBS (Fig 1). The boundary between the Skagerrak-northern Kattegat sub-region (SK1) and the southern Kattegat-northern Belt Sea sub-region (KB1) was based on the proposed boundaries for harbor porpoise management units at 56.95°N latitude [39]. The border between KB1 and BES2 sub-regions was defined in a previous study [32]. The boundary between the southern Belt Sea (BES2) and Inner Baltic Sea (IBS) sub-regions was based on the proposed boundaries for management units at 13.5°E longitude [39]. We analyzed samples from either by-caught or stranded individuals from the WBS region (n = 4) and Iceland (n = 3), and from samples originating from our focus areas: NOS (n = 6), SK1 (n = 5), KB1 (n = 6), BES2 (n = 10), and the IBS (n = 10) (Fig 1) [32]. A detailed sample information table is available in the Supporting information (S1 Table). We genotyped all samples at ddRAD-seq-derived SNP positions, as well as microsatellite loci to calculate population statistics and infer population structure using spatial principle component analysis (sPCA) and Bayesian clustering. In addition, we correlated mitochondrial haplotype data with specific genotype cluster assignments.

None of the sampling was performed on live specimens, nor has any live harbor porpoise specimen been targeted by any activity related to this study. All sampling was performed on carcasses by the respective national authorities allowed or even obliged to sample harbor porpoise carcasses. Specifically, Icelandic samples originate from the Marine Research Institute in Iceland, a governmental institute that does not require a special permit. German samples originate from the two institutes responsible for the collection of harbor porpoise carcasses at German coasts, i.e., the Institute for Terrestrial and Aquatic Wildlife Research, University of Veterinary Medicine Hannover, for Schleswig-Holstein and Deutsches Meeresmuseum in Stralsund for Mecklenburg-Vorpommern (permissions granted by the respective federal ministries for environmental affairs). Polish samples originate from Hel Marine Station, University of Gdansk, holding the permission to collect carcasses at the Polish Baltic coast. Swedish samples originate from the Swedish Museum of Natural History (SMNH) which is an official national institute holding a permission to collect carcasses for necropsy and sampling of various tissues, including harbor porpoises. Western Baltic Sea samples originate from Istanbul University, which does not require any specific permission to hold genetic samples of harbor porpoises from the Turkish coast of the Black Sea.

### Molecular methods (extraction, sequencing, amplification)

#### DNA isolation

We extracted total genomic DNA from approximately 25 mg of tissue (skin or muscle) from samples stored at -20°C (frozen or stored in ethanol) using the NucleoSpin Tissue Kit (Macherey-Nagel, Germany) following the manufacturer’s recommendations. We measured the DNA concentration using a NanoDrop 1000 (Thermo Scientific, USA). Using the Agilent 2200 TapeStation with the Genomic ScreenTape System (Agilent Technologies, USA), we additionally assessed sample quality and quantity.

#### ddRAD-Sequencing

RAD-seq has become one of the most widely used genotyping methods in population genomics studies of non-model organisms including cetaceans [40,41]. It combines reduced representation library construction, achieved through restriction enzyme (RE) digestion of genomic DNA at conserved sites, and Next Generation Sequencing (NGS) methods. Traditional RAD-seq uses a single RE digest coupled with secondary random fragmentation to generate NGS libraries for single-end or paired-end sequencing [42,43]. ddRAD-seq uses a two enzyme double digest followed by a more precise size selection step which allows greater control of the fraction of regions represented in the final library and ensures better reproducibility [44]. In this study, we used a modified ddRAD-seq approach (see below) [45].

The ddRAD-tag libraries were prepared from total genomic DNA of 49 individuals (five were excluded from this analysis) using the restriction enzymes *Pst*I (a rare cutter) and *Msp*I (a common cutter) by a commercial sequencing company (LGC Genomics, Berlin). Briefly, the DNA samples were normalized and simultaneously digested with both enzymes. This step was followed by adapter ligation, where the *Pst*I adapter contained a unique sample barcode. The reaction mix clean-up using polyethylene glycol (PEG) precipitation was followed by an amplification step to add the flow cell binding sites. This step included a concurrent reduction of the amount of fragments to be sequenced by elongating one of the two PCR primers by two bases [45]. The individual samples were then pooled and cleaned using QIAgen PCR purification kit (Qiagen, Germany). Fragments between 250 and 500 bp were excised from a low-melting point (LMP) agarose gel following electrophoresis. Size-selected fragments were purified using the QIAquick gel extraction kit (Qiagen, Germany).

The libraries were sequenced on one lane of the Illumina HiSeq 2000 platform (Illumina Inc., USA) with the 100 bp paired-end read module. Raw Illumina reads were processed using the *Casava* v. 1.8.2 software (Illumina Inc., USA). Samples were de-multiplexed with inline barcodes using LGC-developed software and clipped to remove Illumina TruSeq™ adapters and inline barcode remnants of all reads. Reads shorter than 20 bases were discarded; the remaining paired read was stored in a separate FASTQ file for single reads. FastQC reports (http://www.bioinformatics.bbsrc.ac.uk/projects/fastqc/) containing read quality metrics were generated for all FASTQ files.

#### Microsatellite genotyping

All samples genotyped using ddRAD-seq were compared to thirty-three samples previously genotyped [32] at 13 polymorphic microsatellite loci: PPHO104, PPHO130, PPHO131, PPHO137, PPHO142 [46], lgf-1 [47], EV94, GATA053 [48,49], KWM12a [50], Tex Vet3, Mk6, Mk8, and Mk9 [51,52]. Following the same protocol, we genotyped additional samples from the WBS region (n = 4), Iceland (n = 3), the BES2 region (n = 2) and the NOS region (n = 2) in the same laboratory to complement the existing samples from the NOS, SK1, KB1, BES2 and IBS regions (S1 File; [32]). Six previously genotyped samples were genotyped again to ensure that allele calling was consistent between studies. The repeatability in genotyping across studies was 97% (i.e., 3% of the alleles were called differentially in [32], relative to the present study).

#### Mitochondrial DNA analysis and sexing

For the same set of eleven additional samples described above, we amplified the 5’ end of the mitochondrial Control Region using primers ProL and DLH and following a previously published protocol [32,46]. Using the Antarctic phosphatase (NEB, USA) protocol according to the manufacturer’s recommendations, we enzymatically purified the reaction products. Purified fragments were processed with the BigDye Terminator v3.1 Cycle Sequencing Kit (Applied Biosystems, Foster City, USA) and sequenced on an ABI PRISM 3130 Genetic Analyzer (Applied Biosystems). Control Region Sequences were aligned using Bioedit v. 7.2.5 [47]. We defined mitochondrial haplotypes based on a comparison of a 414 bp sequence to haplotype PHO1 (Genbank ID: Y13872; S2 File). A mitochondrial haplotype network for all samples genotyped by ddRAD sequencing, including mtDNA information on 33 samples already typed by [32], was constructed using TCS 1.2.1 with default parameter settings [48]. Samples of previously unknown gender were sexed using PCR and the ZFX and SRY-specific primers and cycling conditions as described previously [49].

### ddRAD-seq data analyses

We processed the sequenced data and analyzed reads from all individuals using several programs from the *Stacks* v. 1.35 software package for analyzing RAD-seq data [50,51]. First, we filtered for read quality and trimmed the de-multiplexed paired-end reads to a length of 85 bp using the *Stacks* program process_radtags. We concatenated the two paired-end read files into one common FASTQ file per individual. By running the three *Stacks* components (*ustacks*, *cstacks*, and *sstacks*), we identified the alleles in our populations set. The *ustacks* program aligns short sequence reads into matching stacks from which loci are formed and SNPs detected at each locus. We tested several combinations of parameter settings. For the final data analyses, the minimum depth of coverage required to create a stack and the maximum distance (in nucleotides) allowed between stacks were both set to 3; the removal of highly repetitive RAD-tags was enabled. To increase heterozygote calls, we enabled the bounded-error SNP calling model (upper bound: 0.01). A catalog of all loci across all individuals was created with the *cstacks* program with two mismatches allowed between loci when building the catalog. The *sstacks* program then matched loci from each individual back to the catalog. The *rxstacks* program, which makes corrections to haplotype calls in individual samples based on data from a population of samples, can also filter out catalog loci that have poor coverage or high sequencing error, and thus a low log likelihood of being correct. We filtered out catalog loci that had values below the minimum log likelihood of -20. We then used the *rxstacks* output to rebuild the catalog with *cstacks* and re-matched the reads to the catalog with *sstacks*. Loci were retained if they were present in six out of seven sub-regions, in 80% of all individuals per sub-region, if the depth of coverage at each locus was equal or higher than 10 reads per locus in every individual, and the minimum log likelihood for a locus was -10. To generate the subset of SNPs used in our Bayesian clustering analyses, a locus was retained if it was present in 97% of all 44 individuals (i.e., 42 individuals) with a minimum coverage of 6 in every individual and a log likelihood value of -10. The populations program outputs basic population statistics and also enables output in several common file formats for downstream population genomics or phylogenetic analyses, such as the *Structure* format. The number of SNPs used in Bayesian clustering and spatial PCA analyses (1874 and 2872, respectively) falls into the ranges reported in other population genomics studies using the RAD-seq approach [52–54]. Detection power of SNPs has been previously evaluated by simulation [55]. Among the simulated scenarios there, sample size n = 10 and FST = 0.01 most closely resemble our study. For 75 SNP loci (maximum value evaluated in [55]), detection power was at least 0.32. As we have an about 25x to 40x higher number of SNPs (1874 or 2872 instead of 75) and as power scales with number of loci [55], discrimination power in our study is expected to be considerably higher.

### Population statistics and differentiation analyses

#### Measurements of genetic diversity

For the SNP marker-set, the populations program of *Stacks* outputs basic population genetics statistics for all positions and variant (polymorphic) positions, which include average frequency of the major allele, observed and expected heterozygosity. To correct for differences in sample size, we also subsampled all sub-regions to 3 randomly selected individuals per sub-region. For the microsatellite marker-set we estimated the mean observed and expected heterozygosities for all populations, as well as the 13 microsatellite loci across all populations, and tested for departures from Hardy-Weinberg equilibrium with *Arlequin* 3.5 [56].

#### AMOVA

We computed pairwise F_ST_ comparisons between populations from the set of 13 microsatellite loci and SNP loci that passed filtering criteria in *Arlequin* 3.5 (allowed level of missing data: 15%). For all regions and the NOS to IBS sub-regions, 672 and 870 SNP loci, respectively, were used for the analysis. *Stacks* reports nucleotide loci as ‘unknown’, if it cannot distinguish between a heterozygous and a homozygous state. We performed relevant significance tests using a Holm-Bonferroni correction [57]. We conducted the analysis of molecular variance (AMOVA) for both the SNP and the microsatellite marker-sets in *Arlequin* 3.5. For the mitochondrial haplotype marker-set, we conducted AMOVA for the Φ_ST_ and F_ST_ indices. The hierarchy of the analysis, which was performed on the samples from all regions, and the North Sea to Baltic samples, was chosen based on geographic regions (and sub-regions; in parentheses): (i) Western Black Sea (WBS), (ii) North Atlantic (Iceland, NOS), and (iii) Baltic Sea (SK1, KB1, BES, IBS). The same analysis was repeated for the NOS to IBS sub-region samples.

#### sPCA

To model the population genetic structure without *a priori* population assignment, we used spatial principal component analysis (sPCA) implemented in the R package *adegenet*, using our geo-referenced dataset of 2872 SNPs and the microsatellite marker-set [58]. This approach is highly suitable for analyses of complex or cryptic genetic structures because it does not require assumptions of Hardy-Weinberg and linkage equilibria. We conducted sPCA analyses on the entire dataset (all regions), and then specifically focused on the NOS to IBS sub-regions (eastern North Sea and Baltic Sea, cf. Fig 1). Spatial information was provided by converting ETRS89 geographic coordinates to curvilinear orthogonal coordinates with reference to its centerline as described in [59] using ArcGIS 10.2.1 software. Briefly, a centerline was drawn from the Black Sea to the Baltic Sea. Curvilinear coordinates were calculated as the shortest distance from a sampling point to the line, and the distance from this intercept to the beginning of the line. All maps were created using ArcGIS software by ESRI. ArcGIS and ArcMap are the intellectual property of ESRI and are used herein under license (2013 ESRI, Redlands CA, USA). sPCA eigenvalues were tested for global structures, which corresponds to positive spatial autocorrelation between individuals, such as patches, clines and intermediates, and local spatial structure, which indicates strong genetic differences between neighbors, separately for each analysis using Monte Carlo tests (10,000 permutations). This test was performed separately for each data set (i.e., entire data set and eastern North Sea/Baltic Sea subset). For the NOS-IBS region, we tested for isolation-by-distance using the Mantel test as implemented in *adegenet*.

#### Bayesian clustering

In addition, we used the Bayesian algorithms implemented in the *Structure* software package v. 2.3.4 to explore the population genetic structure based on our SNP marker-set [60–62]. After applying more stringent filtering parameters in the *Stacks* pipeline, we used 1874 SNP loci typed in at least 97% (42 out of 44) of the individuals as input. From each RAD-tag locus, only one SNP was chosen to avoid using loci in tight linkage in *Structure* analyses [59]. We conducted a separate analysis of the individuals from the NOS to IBS sub-regions following the same protocol. To streamline batch mode analyses of population structure by setting up multiple iterations for various values of parameter *K* (assumed number of genetic clusters), we used the freely available program *StrAuto* (www.crypticlineage.net/pages/software.html), modified for parallel processing (http://genome.smcm.edu/emersonLab/software.html). For analyses, we ran 100,000 burn-in iterations and 200,000 MCMC repetitions, with 15 replicates for each value of *K*, which ranged from 1 to 8, and the standard admixture model. Lambda, the Dirichlet parameter for estimating allele frequencies, was inferred to be 0.36. The StrAuto output builds a zip archive containing all result files which we uploaded to *Structure Harvester* (http://taylor0.biology.ucla.edu/structureHarvester/) [63], a program for visualizing *Structure* output and implementing the Evanno method [64]. We chose optimal values of *K* based on the Evanno Δ*K* values. To align multiple replicates of our data sets and facilitate the interpretation of clustering results, we used the computer program *Clumpp* (CLUster Matching and Permutation Program) [65]. We visualized *Structure* results with *distruct* v. 1.1 (http://www.stanford.edu/group/rosenberglab/distruct.html).

For the microsatellite marker-set, we used the same *Structure* parameter settings, except for the allele frequencies parameter lambda, which was kept at the default value of 1. To improve the performance of *Structure* on the microsatellite marker-set with a weak signal for population structure, which may be the case in datasets with few loci or individuals, we used the LOCPRIOR model.

## Results

### DNA quality and sequencing output

The analysis of DNA quality revealed marked differences in DNA integrity between samples, as would be expected from opportunistically sampled tissue (from strandings and by-catches). Fragment lengths with highest intensities per sample ranged from 665 to over 19 thousand bp, with the mean length of fragments for all samples being 9,923 ± 4,782 bp (standard deviation, SD).

One lane of sequencing produced over 296 million de-multiplexed raw reads (or over 148 million raw read pairs) from 49 individuals (5 were not used in this analysis). The average number of adapter-clipped read pairs per individual was 3,023,030 ± 949,813 (SD), with the lowest numbers just above 1.4 million and the highest 5.9 million read pairs per individual. Typically, samples of low DNA quality had a lower number of read pairs. The percentage of reads removed by quality and ambiguous RAD-tags filters in the process_radtags program, was 13.4 and 9.9, respectively, resulting in over 227 million, or 76.7%, of retained reads.

While the *Stacks* catalog contained 847,321 loci, the average number of unique RAD-tag loci per individual was 370,725 ± 63,309 (SD). On average, 29,263 or 8% of those loci were polymorphic (i.e., heterozygous). After applying stringency filters in the populations program to ensure that the loci were present in 80% of individuals from each of the seven sub-regions with sufficient coverage, we retained a set of 2872 loci. For Bayesian clustering, we used a subset of 1874 loci, which were present in 42 out of 44 individuals.

### Harbor porpoise population statistics

For the SNP loci that were polymorphic in at least one of the populations, the average major allele frequency was 0.94 in all sub-regions but WBS (0.97) (Table 1), and the respective average observed heterozygosity ranged from 0.052 (WBS) to 0.102 (Iceland). The lowest level of genetic diversity was found in Black Sea region, which also had the lowest percentage of polymorphism among the 2872 shared polymorphic loci of the entire data set, and the highest values in the Icelandic sample set. Within the North Sea and Baltic sub-regions, samples from IBS had the lowest average observed heterozygosity. The highest percentages of polymorphic loci were found in the BES2 and IBS populations (above 45% of the 2872 loci were polymorphic within these two populations). However, when corrected for sample size, the average observed heterozygosity and the percentage of polymorphic loci were lowest in the BES2 region. The average observed heterozygosities for the microsatellite marker-set were again lowest for the Black Sea population (0.682) and highest for the Icelandic population (0.917; Table 2). When considered separately, all populations were in Hardy-Weinberg equilibrium (HWE). However, when testing each locus across all populations, significant departures from HWE were observed at three microsatellite loci and two additional loci were marginally significant (S2 Table).

**Table 1:** Summary genetics statistics calculated by the *Stacks populations* program for 2872 variant (polymorphic) loci and all loci from all samples (top). and standardized samples (three randomly selected individuals per sub-region; N = 3).

	N	Private	% Poly. Loci	P	H_*OBS*_	H_*EXP*_	F_IS_	% Poly. Loci	P	H_*OBS*_	H_*EXP*_	F_IS_
All samples			Variant loci					All loci				
WBS	4.0	132	13.96	0.9659	0.0516	0.0471	0.0050	0.10	0.9998	0.0004	0.0003	0
Iceland	3.0	195	26.68	0.9398	0.1023	0.0878	0.0052	0.19	0.9996	0.0007	0.0006	0
NOS	5.2	209	36.52	0.9391	0.0987	0.0932	0.0117	0.26	0.9996	0.0007	0.0007	0.0001
SK1	4.2	177	31.64	0.9397	0.0994	0.0901	0.0064	0.23	0.9996	0.0007	0.0006	0
KB1	5.5	253	38.91	0.9391	0.0999	0.0939	0.0086	0.28	0.9996	0.0007	0.0007	0.0001
BES2	8.9	291	46.05	0.9387	0.0975	0.0951	0.0100	0.33	0.9996	0.0007	0.0007	0.0001
IBS	8.4	247	45.18	0.9391	0.0945	0.0942	0.0177	0.32	0.9996	0.0007	0.0007	0.0001
Standardized samples			Variant loci					All loci				
WBS	3	178	16.27	0.9557	0.0686	0.0597	0.0053	0.11	0.9997	0.0005	0.0004	0
Iceland	3	276	33.94	0.9233	0.1301	0.1116	0.0069	0.24	0.9995	0.0009	0.0008	0
NOS	3	240	31.91	0.9235	0.1241	0.1088	0.0118	0.22	0.9995	0.0009	0.0008	0.0001
SK1	3	247	33.12	0.9238	0.1295	0.1104	0.0051	0.23	0.9995	0.0009	0.0008	0
KB1	3	212	32.72	0.9252	0.1280	0.1085	0.0040	0.23	0.9995	0.0009	0.0008	0
BES2	3	162	29.29	0.9284	0.1191	0.1012	0.0041	0.21	0.9995	0.0008	0.0007	0
IBS	3	226	32.57	0.9248	0.1282	0.1083	0.0033	0.23	0.9995	0.0009	0.0008	0

N–average number of individuals genotyped at each locus; Private–number of variable sites unique to each population; % Poly. Loci–percentage of the polymorphic loci found polymorphic within a particular population; P–average frequency of the major allele; *H*_OBS_−average observed heterozygosity per locus; *H*_EXP_−expected heterozygosity: F_IS_−average Wright’s inbreeding coefficient.

**Table 2:** Average observed and expected heterozygosities per population for microsatellites.

Population	Genotypes	H_*OBS*_	H_*EXP*_
WBS	4	0.682	0.672
Iceland	3	0.690	0.829
NOS	6	0.713	0.778
SK1	5	0.723	0.774
KB1	6	0.767	0.816
BES	10	0.875	0.867
IBS	10	0.917	0.828

### Harbor porpoise population structure

Pairwise F_ST_ comparisons between populations were calculated from the SNP and microsatellite loci that passed filtering criteria. Highest F_ST_ values were calculated for pairwise comparisons that included the WBS region (average SNP F_ST_ = 0.20, average microsatellite F_ST_ = 0.13). For the SNP data, F_ST_ values were 0.011 and -0.014 between adjacent sub-regions like NOS:SK1 and SK1:KB1, respectively (Table 3). In comparison, these values were both higher (0.034 and 0.027, respectively) for the microsatellite set. Comparisons between the Baltic Sea and adjacent regions yielded comparatively low F_ST_ values for both datasets.

**Table 3:** Pairwise F_ST_ values for the SNP marker-set (672 SNPs; above the diagonal) and for the microsatellite marker-set (below the diagonal). Values in bold are significant following the Holm-Bonferroni correction at an experiment-wise error rate of α = 0.05.

	WBS	Iceland	NOS	SK1	KB1	BES2	IBS
WBS		0.194	0.194	0.240	0.180	**0.187**	**0.207**
Iceland	0.149		-0.014	-0.038	-0.002	-0.002	-0.011
NOS	0.095	0.036		0.011	0.012	**0.034**	0.027
SK1	0.161	0.100	0.034		-0.014	0.012	0.012
KB1	0.131	0.037	0.024	0.027		-0.006	0.003
BES2	**0.122**	0.038	0.020	0.045	0.000		0.002
IBS	**0.130**	0.035	0.028	0.054	0.008	-0.003	

We conducted a hierarchical AMOVA for populations spanning all seven sub-regions, as well as the North Sea to Baltic Sea area, using the SNP, microsatellite, and mitochondrial haplotype marker-sets. For the WBS to Baltic Sea regions, the largest source of variation was between individuals within sub-regions (over 91%), followed by variation among regions when considering nuclear markers (Table 4). For the NOS to IBS sub-regions, the largest source of variation was again between individuals within sub-regions (over 97%). Global AMOVA results calculated as a weighted average over loci yield similar percentages of variation at all hierarchical levels, which were significant for the SNP marker-set among regions for both the entire data set and the NOS to IBS sub-regions (S3 Table).

**Table 4:** Results of AMOVA for the SNP and microsatellite marker-sets performed for all regions and the North Sea to the Baltic Sea sub-regions.

	SNP marker-set			Microsatellite marker-set		
Source of variation	d.f	Percentage of variation	P-value	d.f	Percentage of variation	P-value
All regions*						
Among regions	2	8.18	0.009	2	4.29	0.020
Among sub-regions within regions	4	0.14	0.241	4	2.15	0.015
Within sub-regions	81	91.68	<0.001^#^	81	93.56	<0.001^#^
North Sea–Baltic Sea**						
Among regions	1	2.81	0.195	1	1.01	0.403
Among sub-regions within regions	3	0.13	0.125	3	1.67	0.089
Within sub-regions	69	97.06	<0.001^#^	69	97.31	0.017^#^

^#^Significance of F_ST_ among all sub-regions (across regions)

*AMOVA based on 672 SNPs and 13 microsatellites

**AMOVA based on 870 SNPs and 13 microsatellites

AMOVA of conventional F_ST_ indices from mitochondrial haplotype frequencies yielded sources of variation comparable to the AMOVA based on nuclear markers (Table 5). Here, only the lowest level of hierarchy for all regions was significant. Conversely, AMOVA with the Φ_ST_ index showed that the largest source of variation was among regions (over 67%) when considering samples from the Black Sea to the Baltic. The second largest source of variation was between individuals within sub-regions (over 28%). Both of these results were significant. For the NOS to the Baltic Sea sub-regions, the largest significant source of variation was at the lowest level, between individuals.

**Table 5:** Results of AMOVA for the mitochondrial haplotypes for F_ST_ and Φ_ST_ indices, performed for all regions, and the North Sea to Baltic Sea sub-regions.

	F_ST_			Φ_ST_	
Source of variation	d.f	Percentage of variation	P-value	Percentage of variation	P-value
All regions					
Among regions	2	12.60	0.072	67.42	0.007
Among sub-regions within regions	4	7.41	0.084	4.07	0.067
Within sub-regions	37	79.99	0.004^#^	28.52	<0.001^#^
North Sea–Baltic Sea					
Among regions	1	17.16	0.199	26.78	0.201
Among sub-regions within regions	3	1.21	0.377	6.29	0.069
Within sub-regions	32	81.63	0.080^#^	66.94	0.009^#^

^#^Significance of F_ST_/Φ_ST_ among all sub-regions (across regions)

**Fig 2:**
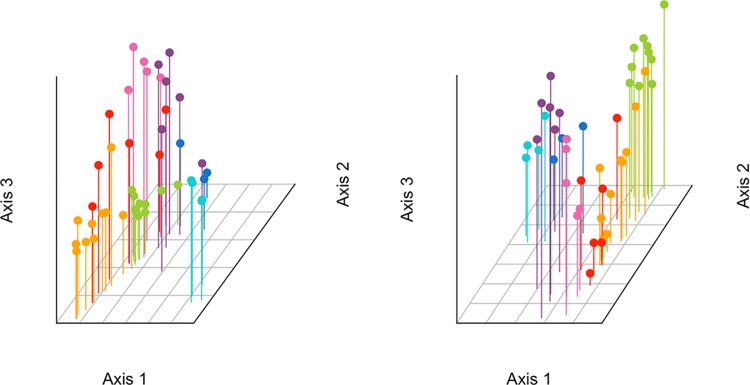
Three-dimensional plots using the first three eigenvalues of sPCA for the SNP (left) and microsatellite (right) datasets for all sub-regions. Values along axes 1, 2 and 3 represent lagged principal scores for each genotype relative to the eigenvalue. Colors denote sampling regions or sub-regions: WBS–light blue, Iceland–dark blue, NOS–purple, SK1 –pink, KB1 –red, BES2 –orange, IBS–green.

**Fig 3:**
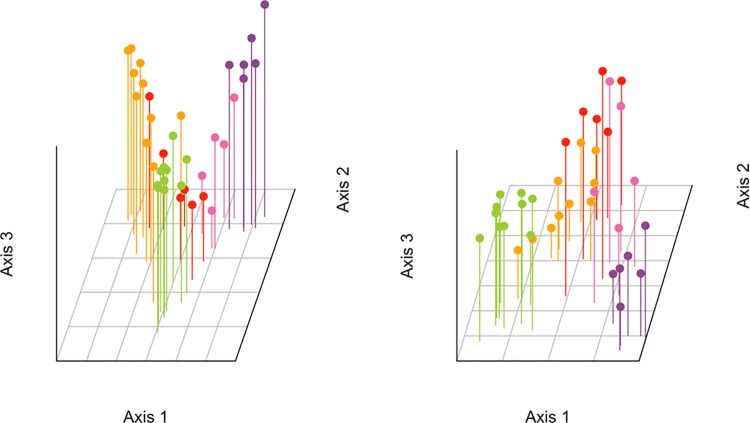
Three-dimensional plots using the first three eigenvalues of sPCA for the SNP (left) and microsatellite (right) datasets for the NOS to IBS subset. Values along axes 1, 2 and 3 represent lagged principal scores for each genotype relative to the eigenvalue. Colors denote sampling sub-regions: NOS–purple, SK1 –pink, KB1 –red, BES2 –orange, IBS–green.

**Fig 4:**
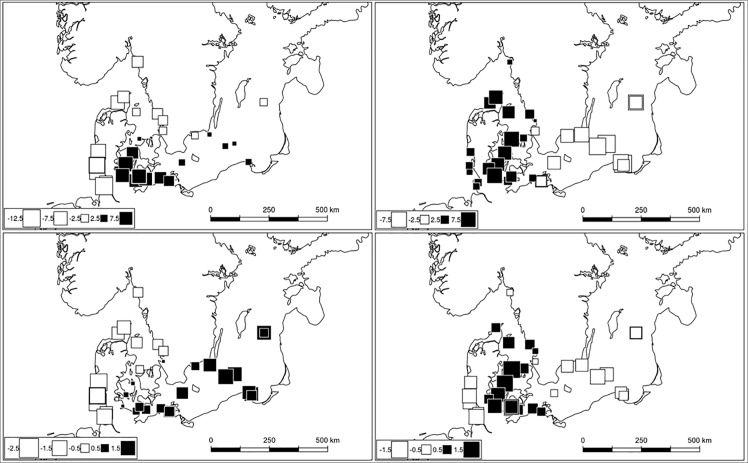
The first (left panels) and second (right panels) global scores of the sPCA of SNP (top panels) and microsatellite (bottom panels) datasets for the NOS to IBS subset. The squares represent the score (white–positive, black–negative) of each genotype and are positioned according to their spatial coordinates on the map of the Baltic Sea and adjacent sub-regions. Map data: ESRI (2013).

We performed sPCA using both nuclear marker-sets on all sub-regions, and those ranging from the NOS to the IBS. Results indicated statistically significant global spatial structures for all combinations of markers and datasets, but no significant local structures (S4 Table). In order to produce three-dimensional plots, we retained the first three eigenvalues from all analyses (S1 Fig). When considering all regions, the WBS individuals were partitioned from the North Sea and the Baltic regions using both the SNP and microsatellite marker-sets along the first global eigenvalue (Fig 2). The NOS samples were separated from the Baltic Sea sub-regions along the second global axis. This separation is clearer in the SNP marker-set. For samples raging from the North to the Baltic Sea, a separation of NOS and Baltic Sea regions along axis 1 was clear using both marker datasets (Fig 3). The separation of the IBS sub-region from BES2 along axis 2 was evident in the SNP marker set. A further separation of BES2 individuals from most of the KB1 and especially SK1 was observed along the third axis. For the NOS to IBS sub-regions, we mapped the genotype scores relative to the first and second axes of the sPCAs based on both marker-sets (Fig 4). We identified a NOS to IBS differentiation pattern, indicative of isolation-by-distance (IBD), with a boundary, however, in the Southern Kattegat for the SNP marker-set. The second sPCA scores differentiated individuals from the KAT/BES and IBS sub-regions in both datasets

We tested for IBD between all regions and the NOS to IBS subset using a Mantel correlation test between the genetic and geographic distance matrices for the SNP and microsatellite marker-sets (9999 permutations). IBD was not significant when combining individuals from all regions for either dataset (SNP marker-set *P*-value = 0.63; microsatellite marker-set *P*-value = 0.73). Apparently, on this scale population divergence is not directly correlated to mere geographic distance. For example, Icelandic samples are assigned to the same cluster as North Sea samples, despite of the geographic distance among them. For the NOS to IBS sample subset, the test based on both marker-sets indicated significant IBD only for the microsatellites (SNP marker-set *P*-value = 0.83; microsatellite marker-set *P*-value = 0.01), while SNPs revealed a more complex genetic structure not directly correlated with distance (see below).

**Fig 5:**
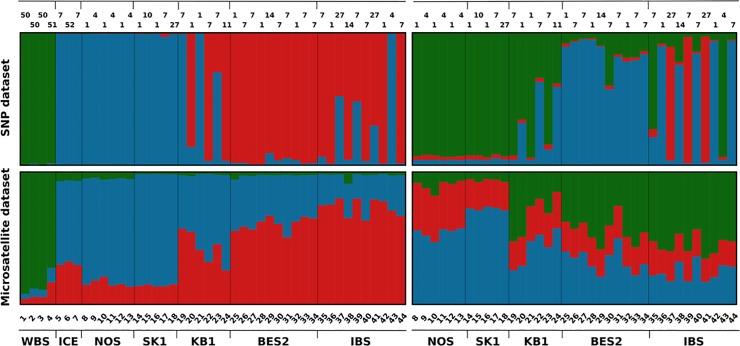
Assignment of individuals into three clusters for all regions (left panels), and for the NOS to IBS sub-regions (right panels) based on *Structure* analyses with SNP (top panels) and microsatellite (bottom panels) marker-sets. The results are grouped by sub-region of origin. Each of the 44 individuals is represented with a vertical column where the coloration is proportional to the individuals estimated membership coefficient in one of the given clusters of genetic similarity. Individuals’ IDs are given below the plots with corresponding mitochondrial haplotypes above the plots.

We also analyzed both nuclear marker sets with the Bayesian clustering algorithm implemented in *Structure*. When considering all regions, we found that the model with three clusters best fit the data for the SNP set (Δ*K* = 31.4), and with 2 clusters for the microsatellite sets (Δ*K* = 21.55) (Fig 5, S5 Table, S2 Fig). Both plots revealed a pattern where the Black Sea population was clearly separated from all the others, at the highest level of structure. In the SNP-based plot, Icelandic samples clustered with the NOS and SK1 samples. Individuals from the BES and IBS sub-regions were assigned to a third cluster. Analysis of the SNP marker-set for NOS to IBS samples yielded the highest Δ*K* values for *K* = 3 and *K* = 5 [63]. For the microsatellites, the inferred value was *K* = 2. Notably, for the SNP marker-set, three individuals from the IBS region cluster separately. For comparative purposes, plots with three clusters are shown in Fig 5. Plots with best *K* values for the SNP marker-set (*K* = 5, NOS to IBS regions) and for the microsatellite marker-set (*K = 2*) are shown in the Supporting Information (S2 Fig).

SNP clusters identified by *Structure* analyses for all samples (WBS to IBS) significantly differed in their mitochondrial haplotype composition (see Fig 5 for assignments). There was a significant over-representation of haplotype PHO7 in the red SNP cluster and a significant overrepresentation of PHO4 in the blue SNP cluster (Table 6). In the SNP-dataset, an individual (No. 43) sampled in the IBS, clustered within the NOS (blue) group and possessed the PHO4 haplotype. Haplotype PHO7 and the nearly ubiquitous haplotype PHO1, separated by a single mutation, were the most abundant ones (S3 Fig). Specific haplotypes were identified for individuals from the Black Sea and Iceland.

**Table 6:** Association between SNP Structure analysis-derived clusters for WBS to Baltic regions and mitochondrial haplotypes (cf. Fig 5 left panels–all samples). The haplotype distribution between the two SNP clusters is significantly different (Χ^2^ = 6.111, p = 0.047 for all haplotypes), due to a significant difference in the occurence of the haplotypes PHO4 and PHO7 (Χ^2^ = 6.111, p = 0.013 for PHO4/PHO7 only).

	Mitochondrial haplotype		
	PHO 4	PHO 7	Other haplotype
Blue SNP cluster	4	5	9
Red SNP cluster	0	11	14

## Discussion

### A population genomics approach to improve population delineation of Baltic harbor porpoises

Using a population genomics approach and a spatially explicit analysis of the genotypic data, we were able to improve the population delineation of harbor porpoises inhabiting the Baltic Sea with adjacent regions–a series of sub-basins that connect this marine ecosystem with the North Sea. We successfully confirmed the separation between the North Sea and the Baltic Sea regions [27]. Our genomic evidence furthermore suggests porpoise sub-populations inhabiting the southern Belt Sea and the Baltic Sea Proper that are genetically distinct from each other. Such a boundary at the Rügen peninsula agrees with a putative boundary separating harbor porpoise management units at 13.5°E longitude, which was proposed based on satellite tracking and acoustic data [39]. In addition, this border is corroborated by differences in skull morphology, segregating the Belt Sea and the IBS porpoises [33]. Even though our sample set was not very large, we achieved good resolution at the level of individuals based on the SNP marker-set and assigned three IBS specimens with high probability to a separate cluster (Fig 5). With our clustering analysis, we were however not able to establish a geographical division within the inner Baltic region, presumably due to so far limited sample size. Future investigations should include a sufficiently large sample set from all seasons to resolve this issue and to account also for potential seasonal movements.

The spatially explicit analysis in this study indicates a separation between the samples from the North Sea and the Baltic Sea individuals. The Skagerrak and northern Kattegat are high-density areas for the harbor porpoise, and correspond to the southern-most distribution of herring (*Clupea harengus*), their main prey species [38,66,67]. *Structure* clustering and pairwise F_ST_ comparisons, however, did not support differences between NOS and SK1. A higher sample number, improved sampling of Danish and Norwegian coastal regions, and consideration of seasonal migrations of porpoises from the Skagerrak would likely resolve these inconsistencies. Prey specialization for pelagic species like herring, and benthic species like cod (*Gadus morhua*) has also been linked to differences in skull morphology between the Skagerrak and the Belt Sea porpoises [33]. A sub-population boundary in the central Kattegat, south of the island of Laesø at 56.95°N latitude has recently been proposed based on satellite tracking studies [38,39]. The genetic differentiation of porpoises from the central Kattegat and northern Belt Sea regions is not as clear as the one between BES2 and IBS. Our spatially explicit analysis suggests a possible separation of porpoises between the Kattegat/northern Belt Sea (KB1), and the southern Belt Sea samples that lies north of Øresund, the Little Belt and in the Great Belt in Danish waters. This finding is in agreement with a tentatively proposed split within BES (BES1 and BES2), and corresponds to the seasonal migrations of porpoises from the Kattegat to the southern part of the Great Belt [32,38].

We also observed frequency shifts of mitochondrial haplotypes between the North Sea and the Baltic regions, as reported previously [32], as well as a significant correlation between haplotypes and the genotypes revealed by the clustering analyses, especially based on the SNP marker-set. As mtDNA and nuclear SNPs are genetically unlinked, such co-occurrence may allow for the identification of migrating individuals, as exemplified with specimen 43, which originates from IBS, but shows affinities to NOS both in the SNPs (green cluster in NOS to Baltic Sea analysis) and the mitochondrial haplotype (PHO4; cf. Fig 5). For the NOS to Baltic Sea, the percentage of genetic variation between sub-regions is much higher for the Φ_ST_-based analyses of mitochondrial haplotypes compared to nuclear the nuclear markers. These observations could indicate maternal philopatry and male-biased gene flow, as has been postulated for harbor porpoises [32].

The genetic differentiation from the North Sea to the Baltic Sea proper is correlated with the geographic distance. The Baltic Sea with its adjacent regions, however, is not a continuous environment such that isolation-by-distance alone is unlikely to explain all genetic differences found in harbor porpoises [23,33,68,69]. It contains gradients in temperature, salinity and depth, and is subdivided by shallow underwater ridges (up to 50 m depth) [69]. In fact, only a small amount of harbor porpoise genetic variation could be attributed to IBD [32]. Similarly, morphological segregations of skull shape do not represent a continuous change in the direction from NOS to IBS [33].

While our study focused on the population differentiation within the Baltic and adjacent regions, we also included two other European harbor porpoise populations from the Western Black Sea and Iceland. The main purpose of including these samples was to test how our experimental and analytical methods position the better-studied European populations. The Black Sea samples representing the subspecies *P*. *phocoena relicta* are most clearly separated from all the other samples based on nuclear as well as private and divergent mitochondrial markers. Historical demographic inferences suggest that the Black Sea descended from the extinct populations that once inhabited the Mediterranean during the glacial and post-glacial period [19]. On the other hand, the samples from Iceland cluster with the North Sea samples and possibly represent the continental shelf ecotype [8].

### Conservation implications

Overfishing, eutrophication, and a drastic decline in marine mammals have been the most prominent changes in the Baltic Sea during the twentieth century [70]. In addition to noise and chemical pollution, gas and oil exploration, and severe winters, by-catch in gill set and drift nets have been the main direct threat to the harbor porpoise [71–74]. The abundance of harbor porpoises in the European Atlantic shelf waters has been estimated at over 375,358 (CV = 0.197), with 19,129 (CV = 0.36) individuals estimated in the regions corresponding to the Skagerrak, Kattegat, and Belt Sea [75]. In a line-transect survey of an area that included the southern Skagerrak region (north of the island of Laesø), the Kattegat and the Belt Sea, porpoise abundance has been estimated at the same order of magnitude, at 40,475 (CV = 0.235) animals [27]. A decline in porpoise abundance has been reported both in the waters of coastal Denmark (the NOS and Skagerrak regions), and in the Belt Sea in the last two decades [75,76]. The abundance of inner Baltic Sea harbor porpoises has been estimated at 599 (CV = 0.57) [28]. Therefore, the status of the inner Baltic Sea porpoises, classified as ‘critically endangered’ by the International Union for Conservation of Nature (IUCN), is of greatest concern [29,30,77]. Such low abundance is even more alarming, since effective population size (Ne) is generally much smaller than the population census size, especially in peripheral populations such as the Baltic [23].

It has been argued [37] that the genetic differentiation of IBS harbor porpoises is too low for them to be considered a separate management unit (MU), as separation should exceed a predefined threshold of divergence [78]. Wiemann *et al*. (2010) subsequently detected a small, but consistent separation of the Baltic proper population from the Belt Sea, and urged for its precautionary acknowledgment as an independent MU [32]. In a recent satellite tracking and acoustic survey, 90% of identified porpoises were west of 13.5°E longitude, proposed as a border between the Belt Sea and IBS MUs [39]. Here, we provide genomic evidence that the IBS population is indeed genetically distinct from the neighboring Belt Sea population and should be managed separately.

The Baltic population has been recognized as threatened in 2002 by the Parties of the Agreement on the Conservation of Small Cetaceans of the Baltic and North Seas (ASCOBANS) that prepared the ‘Recovery Plan for the Baltic Harbour Porpoises (Jastarnia plan)’ [74]. In 2009 and 2012, similar conservation plans were adopted for the North Sea including the Skagerrak, and for the Kattegat, Belt Sea, and the western Baltic, respectively [73,79]. Our results suggest further subtle genetic segregation between the southern Kattegat and Belt Sea animals.

While there is an urgent need to clearly define population borders for the purpose of monitoring and assessment of the conservation status of harbor porpoises, these borders are most likely dynamic. Seasonal migrations of porpoises have been reported for the Skagerrak populations, which migrate westward in the winter towards the southern tip of Norway, for the Kattegat populations, which may migrate southward through the Great Belt in the winter, as well as for the porpoises from the Belt Sea and inner Baltic Sea, which may migrate westward to the Pomeranian Bay and from the Kadet trench into Danish waters, respectively [10,38,76,80]. In addition to including more samples stratified by location and season, reports of seasonal migrations based on acoustic monitoring and satellite tracking to estimate porpoise abundance should be taken into consideration when designing future population genomic studies.

### Population differentiation based on genome-wide SNPs outperforms microsatellite markers

Our study of population differentiation using the ddRAD-seq genotyping-by-sequencing method improved the harbor porpoise population differentiation inferred from microsatellite and mitochondrial control region sequence data [32] and reproduced the correlation between nuclear DNA clustering (microsatellites/SNPs) and certain haplotypes of the genetically unlinked mtDNA. Furthermore, by comparing the results based on the analysis of both nuclear marker types from the same set of harbor porpoise samples, we were able to directly compare the resolution of population structuring acquired by either Bayesian clustering, for which Hardy–Weinberg and linkage equilibra are assumed, or spatial PCA with no *a priori* assignment of individuals to clusters [62,81]. Overall, the spatially explicit method yielded clearer population delineation for both sample sets and for both types of markers. When comparing the resolution achieved using the SNP marker-set or the microsatellite marker-set, assignment of individuals to clusters was improved in *Structure* analyses based on genome-wide SNPs. While microsatellite-based analyses were clearly informative, we obtained a better resolution in spatial analyses using genome-wide SNPs. The ddRAD-seq method provides a genome-wide sampling of loci that is much denser than with microsatellites and yields a much higher number of nuclear markers [82–85]. Consequently, the amount of genetic information per individual is greatly enhanced and the assignment of individuals to populations is facilitated, even for specimens occuring as migrants outside their population of origin.

### Applicability of ddRAD-Seq to opportunistically sampled cetacean samples

ddRAD-seq has become the method of choice in population genomics studies, particularly for non-model organisms [86,87]. An important issue to consider, however, is the quality of genomic DNA, as ddRAD-seq can be limiting in this respect–it requires high-quality genomic DNA [88]. Specifically with regard to harbor porpoise tissue samples it is difficult to acquire or expect ‘fresh’ samples, since most are collected from stranded individuals and a smaller number are from by-catch [89]. Particularly in the case of strandings, tissue is collected from animals in various stages of decomposition with concomitant decreases in DNA quality. As we have seen here, samples of lower DNA quality typically yield a smaller number of unique RAD-tag loci. The application of stringency filters in the bioinformatics pipeline, and increasing the number of samples per study, will yield a smaller dataset for downstream population genomics or phylogenomics analyses set as output. It is therefore critical to consider what level of genomic DNA degradation is acceptable for a sample to be sequenced, to provide a satisfactory RAD-tag output, when sample numbers are increased in future studies.

### Conclusions

In summary, this study demonstrates the feasibility of SNP analysis on opportunistically sampled cetacean samples for population diversity and divergence analysis. This approach should be applied to a larger sample set, such that specimens could be stratified by gender, and incorporate overlaps between populations associated with seasonal migrations observed in abundance estimates, morphological and genetic studies into future study design [39,67]. Using a meaningful and sufficiently large sample set, ddRAD-tag genotyping has the potential to analyze population differentiation with an unprecedented number of loci, which should yield high-resolution power and precision in parameter estimation and population delimitation.

## Supporting Information

S1 Fig Eigenvalue plots of sPCA. (DOCX)

S2 Fig Assignment of individuals into clusters according to best Evanno delta*K* values based on *Structure* analyses with SNP and microsatellite marker-sets. (DOCX)

S3 Fig Mitochondrial haplotype network. (DOCX)

S1 File Microsatellite data. (STR)

S2 File mtDNA data. (FASTA)

S1 Table Detailed sample information table. (DOCX)

S2 Table HWE tests for 13 microsatellite loci over all populations. (DOCX)

S3 Table Global locus-by-locus AMOVA for the SNP and microsatellite marker-sets. (DOCX)

S4 Table Probability (*P*-value) for significant global and local spatial structure for sPCA analyses. (DOCX)

S5 Table Evanno delta*K* values. (DOCX)
