# Supplementary material for: Spatially Explicit Analysis of Genome-Wide SNPs Detects Subtle Population Structure in a Mobile Marine Mammal, the Harbor Porpoise
Source: PLoS One. 2016 Oct 26;11(10):e0162792. doi: 10.1371/journal.pone.0162792 (PMC5082642; doi:10.1371/journal.pone.0162792)
Supplement: S1 Fig — (DOCX) [file pone.0162792.s001.docx]

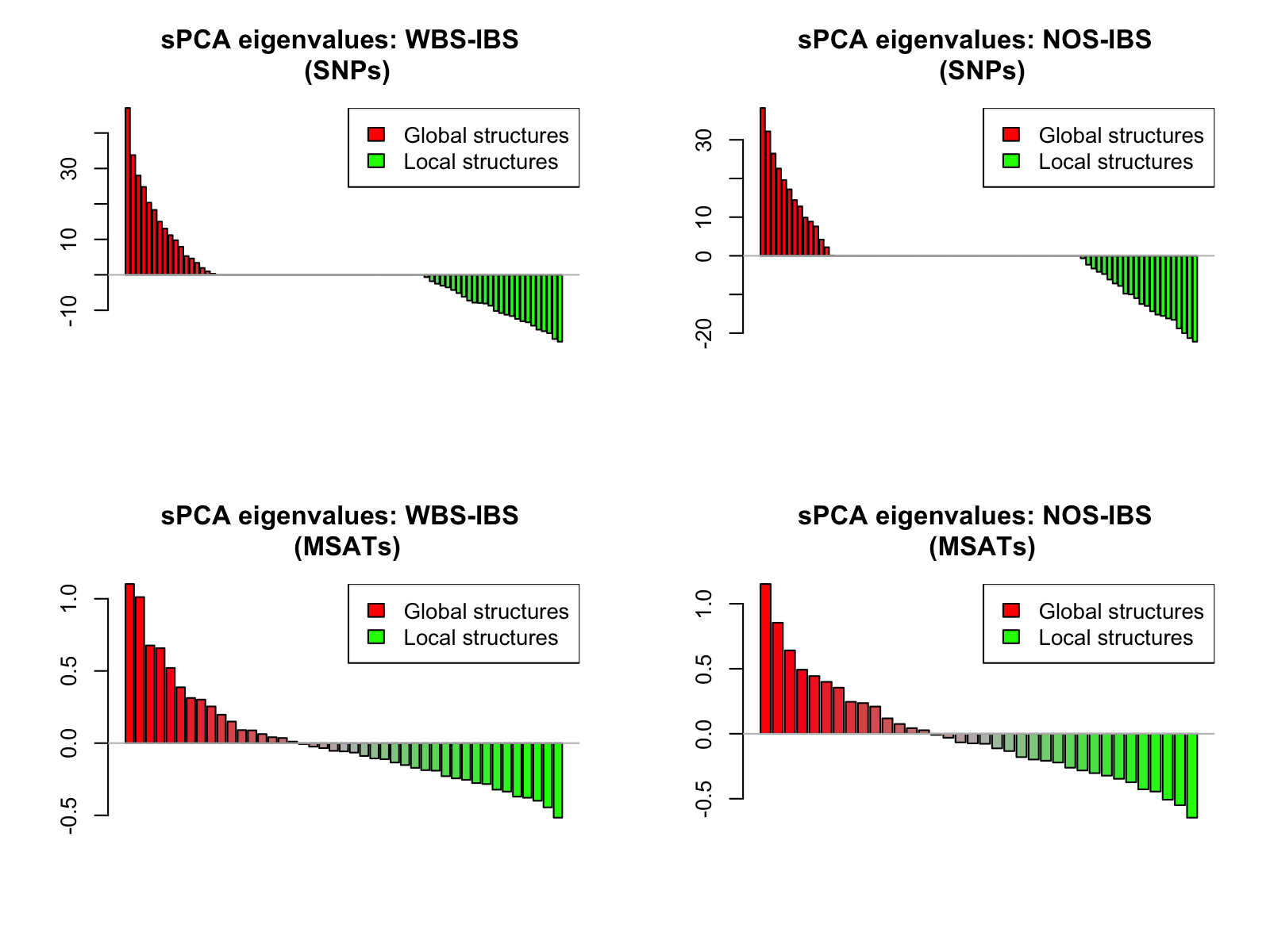


**Figure S1. Eigenvalue plots of sPCA.** Eigenvalue plots of sPCA based on the SNP (top panels) and the microsatellite (bottom panels) datasets for European Seas (WBS-IBS; left panels) and the North Sea to the Inner Baltic Sea sub-regions (NOS-IBS; right panels).
