# Supplementary material for: Spatially Explicit Analysis of Genome-Wide SNPs Detects Subtle Population Structure in a Mobile Marine Mammal, the Harbor Porpoise
Source: PLoS One. 2016 Oct 26;11(10):e0162792. doi: 10.1371/journal.pone.0162792 (PMC5082642; doi:10.1371/journal.pone.0162792)
Supplement: S2 Fig — (DOCX) [file pone.0162792.s002.docx]

**
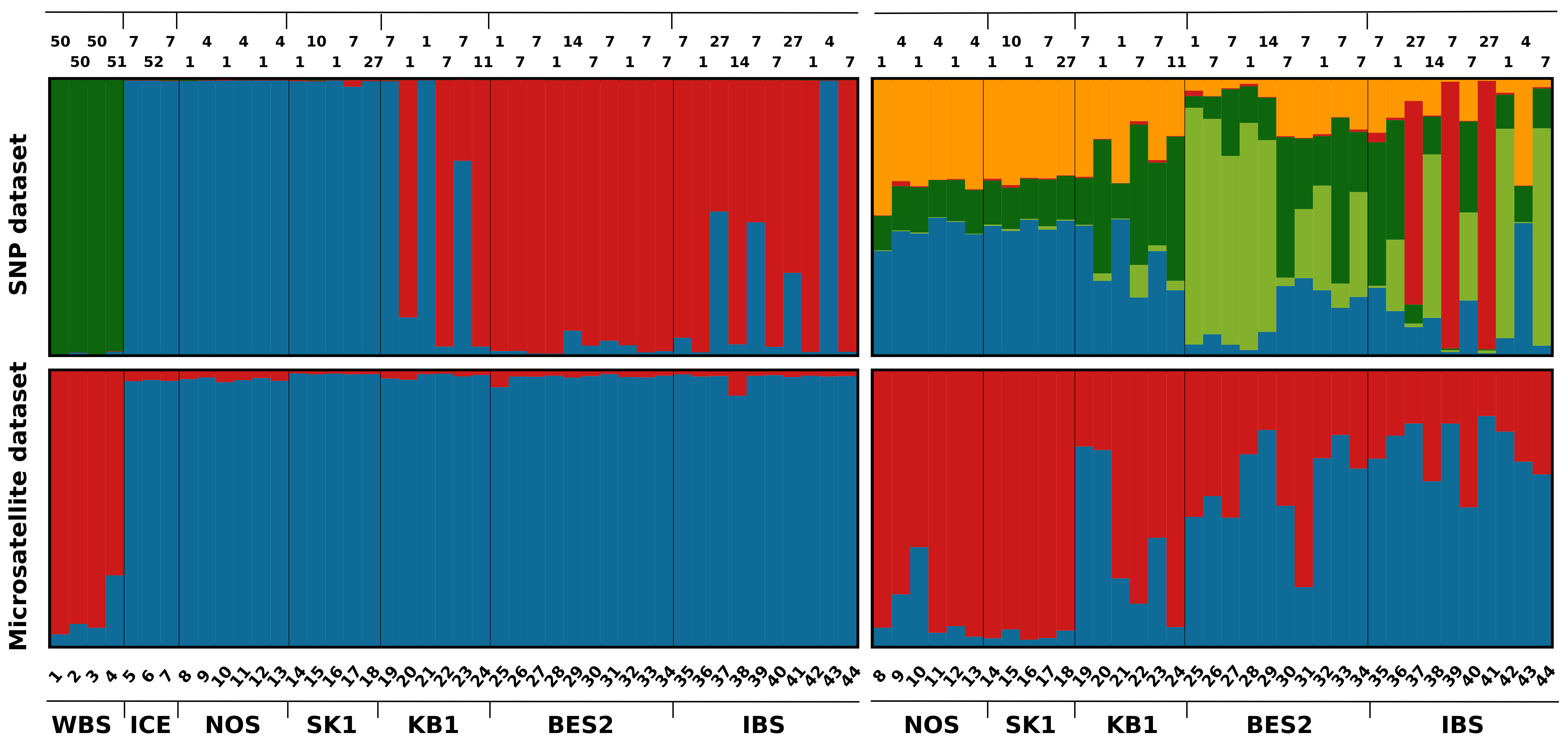
**

**Figure S2. Assignment of individuals into clusters according to best Evanno delta*K* values (Table S5) for all regions (left panels), and for the NOS to IBS sub-regions (right panels) based on *Structure* analyses with SNP (top panels) and microsatellite (bottom panels) marker-sets.** The results are grouped by sub-region of origin. Each of the 44 individuals is represented with a vertical column where the coloration is proportional to the individuals estimated membership coefficient in one of the given clusters of genetic similarity. Individuals’ IDs are given below the plots with corresponding mitochondrial haplotypes above the plots. SNPs – all regions *K*=3; SNPs – NOS-IBS *K*=5; Microsatellites – all regions *K*=2; Microsatellites – NOS-IBS *K*=2.
