# Supplementary material for: Spatially Explicit Analysis of Genome-Wide SNPs Detects Subtle Population Structure in a Mobile Marine Mammal, the Harbor Porpoise
Source: PLoS One. 2016 Oct 26;11(10):e0162792. doi: 10.1371/journal.pone.0162792 (PMC5082642; doi:10.1371/journal.pone.0162792)
Supplement: S3 Fig — (DOCX) [file pone.0162792.s003.docx]

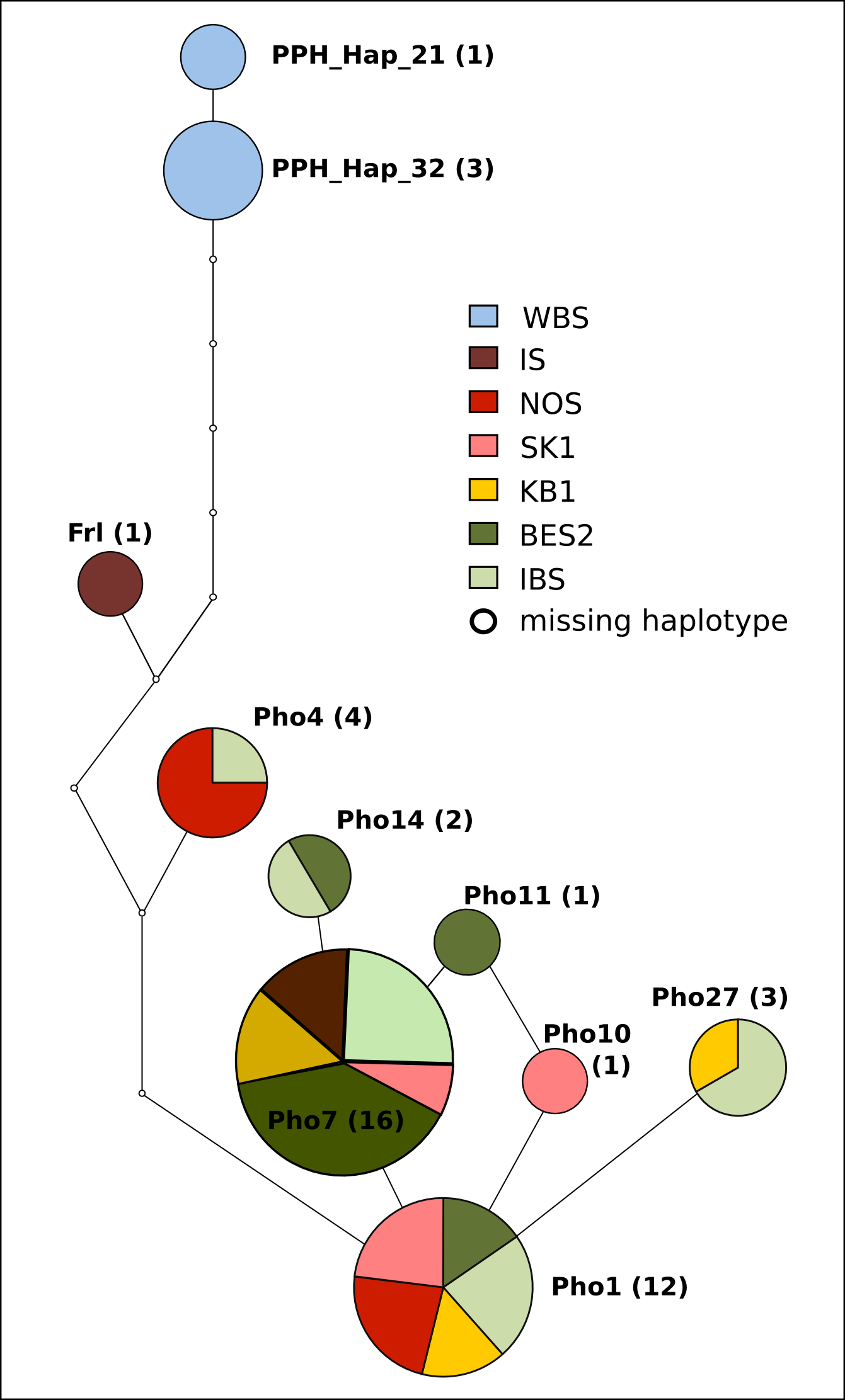


**Figure S3. Mitochondrial haplotype network.** *P. phocoena* mitochondrial haplotypes from the Western Black Sea (WBS), Iceland (IS), North Sea (NOS) in Skagerrak-northern Kattegat (SK1), Kattegat-Belt Sea 1 (KB1), Belt Sea 2 (BES2), and the Inner Baltic Sea (IBS). Circle diameter is proportional to relative frequency of haplotypes. Numbers in parentheses give absolute frequency of occurrence.
