# Supplementary material for: Spatially Explicit Analysis of Genome-Wide SNPs Detects Subtle Population Structure in a Mobile Marine Mammal, the Harbor Porpoise
Source: PLoS One. 2016 Oct 26;11(10):e0162792. doi: 10.1371/journal.pone.0162792 (PMC5082642; doi:10.1371/journal.pone.0162792)
Supplement: S1 Table — (DOCX) [file pone.0162792.s006.docx]

**Table S1. Detailed sample information.**

| **Sample ID** | **Sub-Region** | **Age** | **Sex** | **Sampling Date** | **Bycatch** | **Haplotype** | **ETRS89_X** | **ETRS89_Y** |
| --- | --- | --- | --- | --- | --- | --- | --- | --- |
| 1 | WBS | / | f | Mar-1999 | Y | 50 | 5889933 | 2540761 |
| 2 | WBS | / | f | Jun-2002 | Y | 50 | 5890039 | 2233899 |
| 3 | WBS | / | f | May-2003 | N | 50 | 5839017 | 2227613 |
| 4 | WBS | / | f | May-2003 | Y | 51 | 5832310 | 2244924 |
| 5 | IS | fetus | m | Jan-1996 | / | 7 | 3233759 | 4813580 |
| 6 | IS | / | m | Apr-1992 | / | 52 | 3209607 | 4797929 |
| 7 | IS | fetus | m | Jun-1993 | / | 7 | 3180016 | 4777110 |
| 8 | NOS | adult | f | Sep-1999 | N | 1 | 4214633 | 3511959 |
| 9 | NOS | / | f | Jun-2001 | N | 4 | 4251542 | 3466454 |
| 10 | NOS | adult | f | May-2005 | N | 1 | 4218703 | 3596917 |
| 11 | NOS | adult | f | May-2009 | N | 4 | 4213394 | 3539238 |
| 12 | NOS | adult | f | May-2011 | / | 1 | 4213394 | 3539238 |
| 13 | NOS | adult | m | Apr-2005 | N | 4 | 4243669 | 3449882 |
| 14 | SK1 | / | f | Aug-/ | Y | 1 | 4301922 | 3807197 |
| 15 | SK1 | / | f | Aug-/ | Y | 10 | 4326990 | 3831258 |
| 16 | SK1 | / | / | Feb-2005 | N | 1 | 4387357 | 3981543 |
| 17 | SK1 | / | m | Feb-2005 | N | 1 | 4472196 | 3758971 |
| 18 | SK1 | / | f | July-/ | Y | 7 | 4381834 | 3766792 |
| 19 | KB1 | / | f | May-2005 | N | 27 | 4495892 | 3686361 |
| 20 | KB1 | / | f | Jun-2005 | Y | 7 | 4495929 | 3730905 |
| 21 | KB1 | juvenile | f | July-/ | Y | 1 | 4445852 | 3656849 |
| 22 | KB1 | / | f | Sep-1998 | Y | 7 | 4365334 | 3592274 |
| 23 | KB1 | / | f | Apr-1997 | Y | 11 | 4394941 | 3652012 |
| 24 | KB1 | / | f | Nov-1998 | Y | 1 | 4365334 | 3592274 |
| 25 | BES2 | / | f | Aug-1997 | Y | 7 | 4335938 | 3543863 |
| 26 | BES2 | juvenile | f | Sep-1997 | Y | 7 | 4379419 | 3473504 |
| 27 | BES2 | juvenile | f | Sep-1999 | Y | 7 | 4296359 | 3525358 |
| 28 | BES2 | juvenile | f | Aug-2001 | Y | 1 | 4322079 | 3495618 |
| 29 | BES2 | juvenile | f | Aug-2000 | N | 14 | 4421643 | 3480004 |
| 30 | BES2 | juvenile | f | Nov-2012 | / | 7 | 4393665 | 3491680 |
| 31 | BES2 | adult | f | Aug-2012 | / | 7 | 4393665 | 3491680 |
| 32 | BES2 | juvenile | f | Sep-2001 | / | 1 | 4521633 | 3472075 |
| 33 | BES2 | juvenile | f | Aug-1999 | N | 7 | 4485118 | 3485507 |
| 34 | BES2 | / | f | July-2000 | N | 7 | 4521633 | 3472075 |
| 35 | IBS | / | f | May-/ | Y | 7 | 4576926 | 3550936 |
| 36 | IBS | juvenile | m | Aug-/ | Y | 1 | 4632885 | 3665962 |
| 37 | IBS | / | f | Aug-2000 | Y | 27 | 4801667 | 3631655 |
| 38 | IBS | / | f | Nov-2000 | Y | 14 | 4762796 | 3620971 |
| 39 | IBS | juvenile | m | Aug-/ | Y | 7 | 4695107 | 3670734 |
| 40 | IBS | / | f | Feb-2002 | Y | 7 | 4872424 | 3546611 |
| 41 | IBS | / | f | Jun-2001 | Y | 27 | 4862547 | 3553293 |
| 42 | IBS | / | m | July-1996 | Y | 1 | 4873872 | 3540052 |
| 43 | IBS | juvenile | f | Oct-2003 | / | 4 | 4927053 | 3808759 |
| 44 | IBS | adult | m | Jan-2004 | / | 7 | 4927053 | 3808759 |
