# Supplementary material for: Spatially Explicit Analysis of Genome-Wide SNPs Detects Subtle Population Structure in a Mobile Marine Mammal, the Harbor Porpoise
Source: PLoS One. 2016 Oct 26;11(10):e0162792. doi: 10.1371/journal.pone.0162792 (PMC5082642; doi:10.1371/journal.pone.0162792)
Supplement: S2 Table — (DOCX) [file pone.0162792.s007.docx]

**Table S2. HWE tests for 13 microsatellite loci over all populations.** Values in bold denote significant departures from HWE.

| **Locus** | **Genotypes** | **Obs.Het.** | **Exp.Het.** | **P-value** |
| --- | --- | --- | --- | --- |
| P104 | 43 | 0.837 | 0.866 | 0.580 |
| P130 | 44 | 0.841 | 0.848 | 0.672 |
| P131 | 44 | 0.841 | 0.826 | **0.005** |
| P137 | 43 | 0.791 | 0.893 | **0.036** |
| P142 | 44 | 0.818 | 0.859 | 0.699 |
| GATA053 | 44 | 0.091 | 0.089 | 1.000 |
| Lgf1 | 44 | 0.795 | 0.875 | 0.296 |
| EV94 | 44 | 0.705 | 0.818 | 0.345 |
| Tex3 | 44 | 0.659 | 0.823 | **0.004** |
| KWM12a | 44 | 0.614 | 0.824 | **0.003** |
| MK6 | 44 | 0.659 | 0.801 | **0.038** |
| MK8 | 44 | 0.818 | 0.818 | 0.612 |
| MK | 44 | 0.864 | 0.884 | 0.057 |
