# Supplementary material for: Spatially Explicit Analysis of Genome-Wide SNPs Detects Subtle Population Structure in a Mobile Marine Mammal, the Harbor Porpoise
Source: PLoS One. 2016 Oct 26;11(10):e0162792. doi: 10.1371/journal.pone.0162792 (PMC5082642; doi:10.1371/journal.pone.0162792)
Supplement: S3 Table — (DOCX) [file pone.0162792.s008.docx]

**Table S3. Global locus-by-locus AMOVA for the SNP and microsatellite marker-sets performed for all regions and the North Sea to the Baltic Sea sub-regions.**

|  | SNP marker-set | | Microsatellite marker-set | |
| --- | --- | --- | --- | --- |
| Source of variation | Percentage of variation | P-value | Percentage of variation | P-value |
| All regions* | | | | |
| Among regions | 7.39 | 0.000 | 4.25 | 0.000 |
| Among sub-regions within regions | 1.05 | 0.366 | 2.21 | 0.014 |
| Within sub-regions | 91.66 | <0.001^#^ | 93.56 | <0.001^#^ |
| North Sea – Baltic Sea** | | | | |
| Among regions | 2.06 | 0.000 | 1.01 | 0.221 |
| Among sub-regions within regions | 0.52 | 0.311 | 1.66 | 0.075 |
| Within sub-regions | 97.41 | 0.003^#^ | 97.33 | 0.015^#^ |

^#^Significance of F_ST_ among all sub-regions (across regions)

*AMOVA based on 672 SNPs and 13 microsatellites

**AMOVA based on 870 SNPs and 13 microsatellites
