# Supplementary material for: Spatially Explicit Analysis of Genome-Wide SNPs Detects Subtle Population Structure in a Mobile Marine Mammal, the Harbor Porpoise
Source: PLoS One. 2016 Oct 26;11(10):e0162792. doi: 10.1371/journal.pone.0162792 (PMC5082642; doi:10.1371/journal.pone.0162792)
Supplement: S4 Table — (DOCX) [file pone.0162792.s009.docx]

**Table S4. Probability (*P*-value) for significant global and local spatial structure for both nuclear marker sets for sPCA analyses of all regions and the NOS to IBS subset**. Monte-Carlo test based on 10000 replicates.

|  | SNP dataset | | Microsatellite dataset | |
| --- | --- | --- | --- | --- |
|  | Global structure (*P*-value) | Local structure (*P*-value) | Global structure (*P*-value) | Local structure (*P*-value) |
| All regions | <0.001 | 0.692 | 0.010 | 0.374 |
| NOS-IBS | <0.001 | 0.989 | 0.034 | 0.328 |
