# Supplementary material for: Spatially Explicit Analysis of Genome-Wide SNPs Detects Subtle Population Structure in a Mobile Marine Mammal, the Harbor Porpoise
Source: PLoS One. 2016 Oct 26;11(10):e0162792. doi: 10.1371/journal.pone.0162792 (PMC5082642; doi:10.1371/journal.pone.0162792)
Supplement: S5 Table — (DOCX) [file pone.0162792.s010.docx]

**Table S5**. **Evanno delta*K* values.** Highest values are bolded.

|  | SNP datasets | | Microsatellite datasets | |
| --- | --- | --- | --- | --- |
| K | All populations | NOS to IBS | All populations | NOS to IBS |
| 1 | NA | NA | NA | NA |
| 2 | 0.61 | 0.33 | **21.55** | **9.81** |
| 3 | **31.24** | 2.18 | 0.62 | 1.07 |
| 4 | 1.08 | 0.30 | 0.85 | 0.77 |
| 5 | 2.71 | **2.60** | 0.07 | 0.32 |
| 6 | 0.55 | 0.29 | 0.44 | 0.12 |
| 7 | 0.22 | 0.02 | 0.17 | 0.88 |
| 8 | NA | NA | NA | NA |

*K* – number of clusters
